# Fcodes update: a kinship encoding framework with F-Tree GUI & LLM inference

**DOI:** 10.1515/jib-2024-0046

**Published:** 2025-03-31

**Authors:** Daniel Pérez-Rodríguez, Roberto C. Agís-Balboa, Hugo López-Fernández

**Affiliations:** Dept. of Telematics Engineering, ESEI-Escuela Superior de Ingeniería Informática, Universidade de Vigo, 32004 Ourense, Spain; Science and Business S.L., Edificio CITEXVI, Rúa Fonte das Abelleiras s/n., Local 41.2, Vigo University Campus, 36310, Vigo, Spain; Neuro Epigenetics Lab, Health Research Institute of Santiago de Compostela (IDIS), Santiago University Hospital Complex, 15706 Santiago de Compostela, Spain; Translational Research in Neurological Diseases Group, Health Research Institute of Santiago de Compostela (IDIS), Santiago University Hospital Complex, 15706 Santiago de Compostela, Spain; Servicio de Neurología, Hospital Clínico Universitario de Santiago, 15706 Santiago de Compostela, Spain; SING Research Group, Galicia Sur Health Research Institute (IIS Galicia Sur), SERGAS UVIGO, 36213 Vigo, Spain

**Keywords:** genealogy, data encoding, family structures, computational anthropology, coefficient of inbreeding

## Abstract

Family structures play a crucial role in personal development, social dynamics, and mental health. Traditional systems for encoding genealogical data, such as Ahnentafel and the Register System, offer methods to document lineage but face limitations, particularly in accommodating horizontal relationships or handling changes in family datasets. Modern computational systems like LINKAGE and PED, while powerful for genetic analysis, lack human readability and are challenging to apply in fields where unstructured, narrative data is common, such as sociology or psychiatry. This paper aims to bridge this gap by enhancing Fcodes, a flexible and intuitive algorithm for encoding kinship relationships that is suited for both manual and computational use. Building on our previous work, we present improvements to the Fcodes core algorithm and command-line interface (CLI), as well as the development of F-Tree, a new graphical user interface (GUI) to streamline the encoding process. Additionally, we introduce a method for estimating the coefficient of inbreeding using Fcodes and explore the application of artificial intelligence, namely large language models (LLMs), to automatically infer family relationships from narrative text. These advancements highlight the potential of Fcodes in a wide range of research contexts, from social studies to genetics and mental health research.

## 1 Introduction

As social creatures, we are part of a complex net of relationships that shape our personal growth, daily decisions, mental health, and overall quality of life. The closer the relationship, the greater its influence; that is why the study of family structures, aside from the biological standpoint, is so important for understanding social dynamics, history, and even epidemics, to cite a few examples.

Registering and analyzing this type of information is complex. The first records of an encoding algorithm date from 1,590, where the Austrian historian Michaël Eytzinger designs an encoding system to document the royal European houses (*Thesaurus Principum Hac Aetate In Europa Viventium*). This system, later known as Ahnentafel, was widely used by genealogists for centuries. It assigns the number one to a reference person, doubling for the father and adding one for the mother. This ascending system preserves certain family structure properties, such as lineage tracing (paternal lines follow powers of two, maternal lines are one less), but it overlooks sibling relationships and requires organizing the data before encoding.

In 1870, the New England Historic Genealogical Society (NEHGS) adopted a descending numbering system known as the Register System. It organizes individuals by generation, assigning offspring numbers based on birth order using Roman numerals. In the next generation, these numbers are converted to Arabic numerals, becoming the progenitor’s identifier. The Register System improves the Ahnentafel by allowing the encoding of the horizontal relationships at the cost of the lineage trace. Kinship relationships depend on the format of the data, not on the codes, as was the case with Ahnentafel.

Following the same lines, the National Genealogical Society (NGS) developed in 1912 the NGSQ system, a descending numbering system very similar to the Register System, with analogous strengths and limitations.

Both descending numbering systems depend on the whole dataset to be established before the encoding process; and both are sensible to changes into the offspring, which are propagated through the whole genealogy forcing the reconstruction of the dataset. Still, the Record System and the NGSQ system are the most popular descendancy-numbering systems nowadays [1].

Over the past half-century, the advancement and widespread adoption of computers have enabled the use of sophisticated methods for encoding kinship relationships. The rise of genetics research led to the creation of several formats, such as LINKAGE, FAM, and PED [2], which integrate genetic data with genealogical information. However, while these encoding systems effectively represent family structures, they are designed primarily for computational use rather than human readability or manual production. This has created a gap between traditional and computational approaches to kinship representation.

We believe that a simple and intuitive encoding algorithm for family structures would greatly benefit fields such as psychiatry, psychology, and sociology, where data is often collected through interviews. In these settings, information is typically unstructured, and family members may be recalled or introduced in a non-chronological order. In this context, we established a twofold objective: (i) first, to design an easy and flexible algorithm for encoding family structures, building a link between manual encoding and the computational processing of data; (ii) second, to provide resources for its use and implementation.

These objectives were partially achieved through our recent publication of the Fcodes method, a straightforward algorithm for encoding kinships [3]. Given the versatility of this algorithm, which can be applied across a broad range of contexts – such as social studies, hospital settings, and mental health research – we prioritized providing foundational functions for managing this new type of data.

In this work, we build on our previous efforts to achieve the twofold objective by enhancing the Fcodes core project and its command-line interface (CLI) and by developing a new graphical user interface (GUI), known as F-Tree, on top of the core project. Additionally, to further demonstrate the usefulness of the proposed kinship encoding method, we present a way to estimate the coefficient of inbreeding and explore the suitability of using two of the most powerful large language models for inferring relationships from different narrative texts and encoding them using the Fcodes method.

## 2 Implementation

Fcodes are codes generated by applying the Fcode encoding algorithm to family relationships. The process starts by selecting a person as the reference point, known as the origin of coordinates (OC). From there, relationships to other family members are mapped using a combination of letters, numbers, and symbols. Letters represent the type of direct relationship (e.g., father, mother, daughter), while numbers indicate birth order (e.g., first son, third sister). In this section, we will explain the Fcode encoding algorithm in detail, along with the structure of an Fcode.

### 2.1 Fcode legend

**Figure 1:**
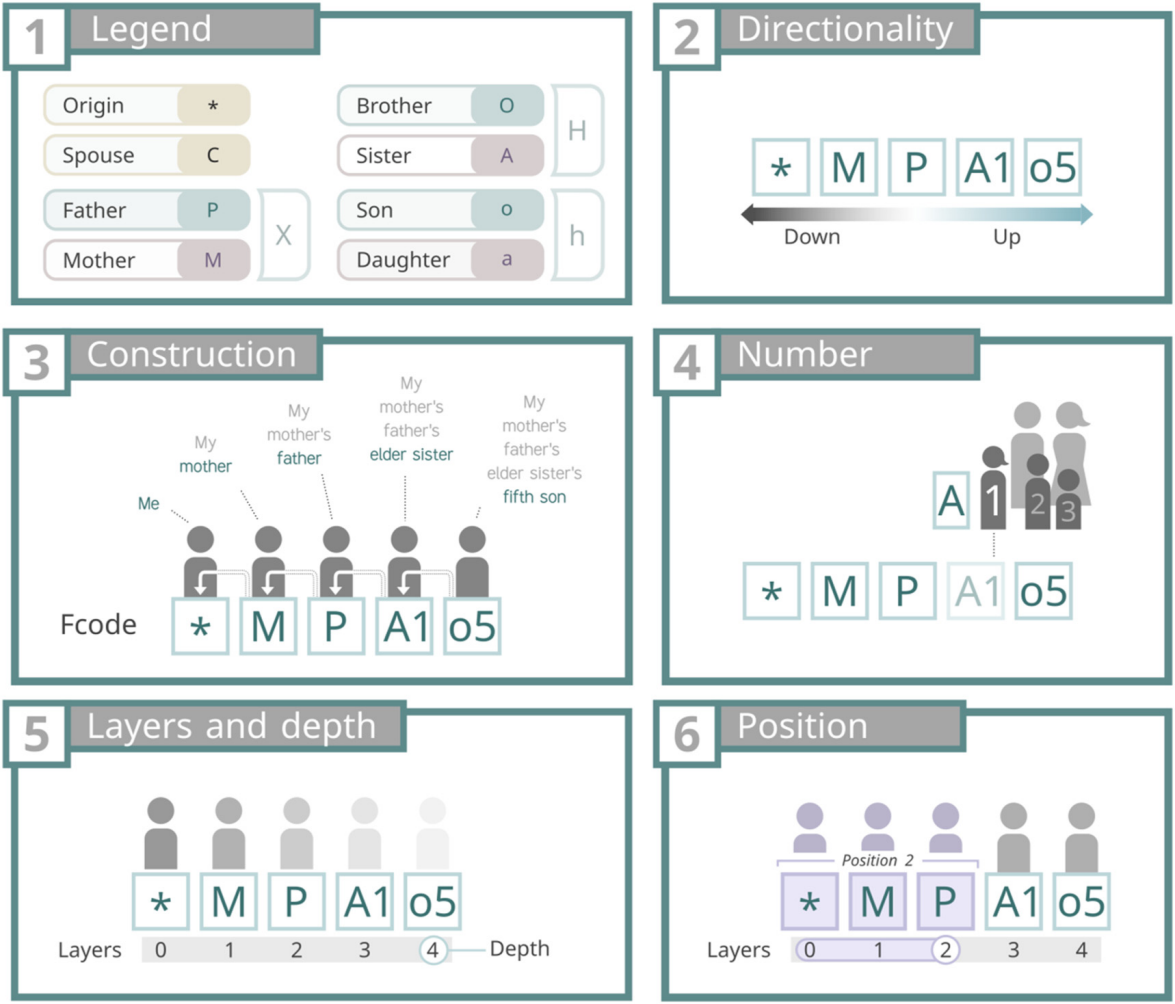
Panel 1 shows the symbols used to encode direct relationships and the origin of coordinates (OC). Panel 2 illustrates directionality, with “down” indicating a direction toward the OC and “up” the opposite. Panel 3 depicts the construction of kinships by adding upper layers and numbers. Panel 4 explains the encoding of birth order with numbers. Panel 5 clarifies the concepts of layers (a combination of a symbol and a number) and depth (the total number of layers). Finally, panel 6 visually represents position, defined as a layer and all downward layers leading to the OC.

As it was previously stated, letters in an Fcode represent the type of direct relationships between its components (Figure 1, panel 1). There are only four types of direct relationships: parenthood, brotherhood, sonship and spousehood; plus the state of being the OC, represented as an asterisk “*”.

While parenthood, brotherhood and sonship have their own encoding (X, H and h, respectively), these concepts were established for the sake of a computational treatment of the encoded data. Therefore, when encoding relationships it is necessary to use the “sexed” versions of these codes, namely: father (P) and mother (M); brother (O) and sister (A); son (o) and daughter (a).

The second part of the legend of an Fcode is the numbers (Figure 1, panel 4), which represent the birth order of the previous letter of the Fcode (see “Layers and depth” in the next section). The numbering must follow these two considerations:

**Consideration 1.** Brotherhood and sonship conditions, sexed or not (O, o, A, a, H, h), must be always numerated. For example: *O3o1a3, *a1o2a2.

**Consideration 2.** Parenthood, sexed or not (P, M, X), and spousehood conditions (C) should also be numerated, but only when they are on the last layer (see “Layers and depth” in the next section). For example: *M2 and *MO4; *P1 and *PO3; or *C3 and *CMP1.

### 2.2 Fcode nomenclature

To define, work and understand the properties of the Fcodes, it is necessary to define a nomenclature. We established six types of conventions: layers, depth, directionality, position, lineage and type.

#### 2.2.1 Layers and depth

In an Fcode, each letter (and its associated number, if present) corresponds to a distinct layer when read sequentially from left to right (see Figure 1, panel 5). Layers are the kinship relationship forming an Fcode; the links between the OC and the person encoded. The layers are numbered starting from the OC, which is assigned layer 0, continuing sequentially up to the final layer, known as the Fcode’s **depth** (Figure 1, panel 5). The greater the depth, the greater the kinship distance between the OC and the encoded person.

#### 2.2.2 Directionality

Fcodes can be read in two directions: left to right (upward) or right to left (downward). In this context, for any given layer, the layer to its right represents the upward direction, while the layer to its left corresponds to the downward direction (see Figure 1, panel 2).

#### 2.2.3 Position

Given an Fcode, the term position refers to a specific layer, and all the downward layers until the OC (see Figure 1, panel 6). For example, given the Fcode “*PMA2O1”, position 0 is *; position 1, *P; position 2, “*PM”, position 3, “*PMA2” and so on.

#### 2.2.4 Lineages and type

A lineage is the code obtained by applying a conversion to an Fcode, where (i) numbers are removed and (ii) letters are replaced with their corresponding counterparts of unknown sex. The upper layer of a lineage is the **type** of the Fcode. For example, the lineage of Fcode “*PO1a1” is “*XHh”, and its type is “h”, as it is the upper layer of its lineage.

### 2.3 Encoding a single person with Fcodes

The process of encoding a person using Fcodes consists of the following steps: (i) Definition of the OC, a person used as a reference to establish kinship relationships. (ii) Connection of the OC with the person to encode by adding layers. The kinship of each letter added refers to the previous position. For example, if we set John as the OC “*”, John’s mother is “*M” and John’s maternal grandmother is “*MM” (the mother “M” of the mother of John “*M”). In this example, numbers are not yet included. If we assume that John’s mother is the eldest among her siblings and John’s grandmother is the fourth in her birth order, the corresponding Fcodes would be: “*”, “*M1”, and “*MM4”.

The connection between the OC and the individual being encoded must be as direct as possible. For example, the Fcode “*PC1o2” is incorrect, as it involves four layers and can be simplified to just two: “*Po2” Since layer “o2” is a child of “*P” and “*PC1”, the more efficient path is through “*P”, rather than taking the longer route via “*PC1”. This topic is covered in depth on section “Fcode patterns”.

### 2.4 Encoding a family

A family can be defined as a group of distinct Fcodes that share the same OC. Considering that each person has been defined by their relationship to the OC, and that all family members share the same OC, the connections between individuals can be traced through this common element, revealing the underlying structure of the family.

We propose a standard format to collect this type of information: the FDATA file. Each line of this file defines a person using two tab-separated columns, the first one storing the Fcode, the second one the name. All the persons in the FDATA file must share the same OC. Empty lines and lines starting with number sign (#) are omitted. Below an example of a valid FDATA file:

# Parents

*   Homer Simpson

*C  Marge Simpson

# Offspring

*o1   Bart Simpson

*a2   Lisa Simpson

*a3   Maggie Simpson

# Grandpas

*P   Abe Simpson

*M   Mona Simpson

We propose the extension “.fdata” to label these types of files.

### 2.5 Fcode patterns

As stated in Section 3.3, the connection between the OC and the last layer of the Fcode must be as direct (i.e. shorter) as possible, avoiding redundancy in references. Making use of the lineages, we can define eight patterns to avoid (see Table 1).

**Table 1:** Wrong Fcode patterns and suggested fixes. A wrong pattern occurs when the kinship of an Fcode is not defined using the shortest path between the origin of coordinates (OC) and the upper layer. This table shows the eight wrong patterns. To detect them, the Fcode must first be converted to its lineage.

Wrong pattern	Right pattern	Fix	Example (wrong)	Example (right)
CC	(loop)	Remove pattern.	*CC	*
hX	X	Remove offspring, change parent to partner.	*a1M3	*C3
HX	X	Remove brother.	*O1M3	*M3
XC	X (sex switched)	Remove partner, switch parent sex (problem with the layer number).	*MC	*P?
Ch	h	Remove partner.	*Ca1	*a1
hH	h	Remove offspring, change brother to offspring.	*a1O2	*o2
HH	H	Remove first brother.	*O2O3	*O3
Xh	H	Remove parent, change offspring to brother. Check if the resulting fcode is the OC.	*Po1	*O1

## 3 Results and discussion

Following the definition of the algorithm, we initially developed a CLI to handle Fcode manipulations [3]. This CLI was further improved to be used as base for the new GUI presented in this work. In addition, we established a strategy to infer the inbreeding coefficient and explored the potential of combining the Fcode method with LLMs to extract family relationships from narrative descriptions automatically.

### 3.1 The Fcode CLI

Searching through large Fdata files manually, while manageable, can be time-consuming. With this limitation in mind we designed a Python-based CLI to simplify working with Fcodes (https://github.com/Dannyzimmer/fcodes). This tool supports the handling of Fdata files, the creation of diagrams and reports, and the random generation of both Fcodes and Fdata for testing purposes.

Since the Fcode CLI was comprehensively covered in our previous paper [3], this discussion will focus on the functions utilized by the GUI.

– **Fcode *search.*** The search command enables locating a specific person within the data. It supports partial matches and allows searches by both Fcodes and names.
– **Fcode *tree.*** This command generates a graphical representation of the Fdata in the form of a tree, which can be saved as a PDF file.
– **Fcode *report.*** This command generates an HTML report in a dictionary-like format from a Fdata file. The report includes an entry for each individual in the Fdata file, detailing their direct relationships with other family members. Each direct relationship is linked to its corresponding entry.

### 3.2 The Fcode GUI: F-Tree

**Figure 2:**
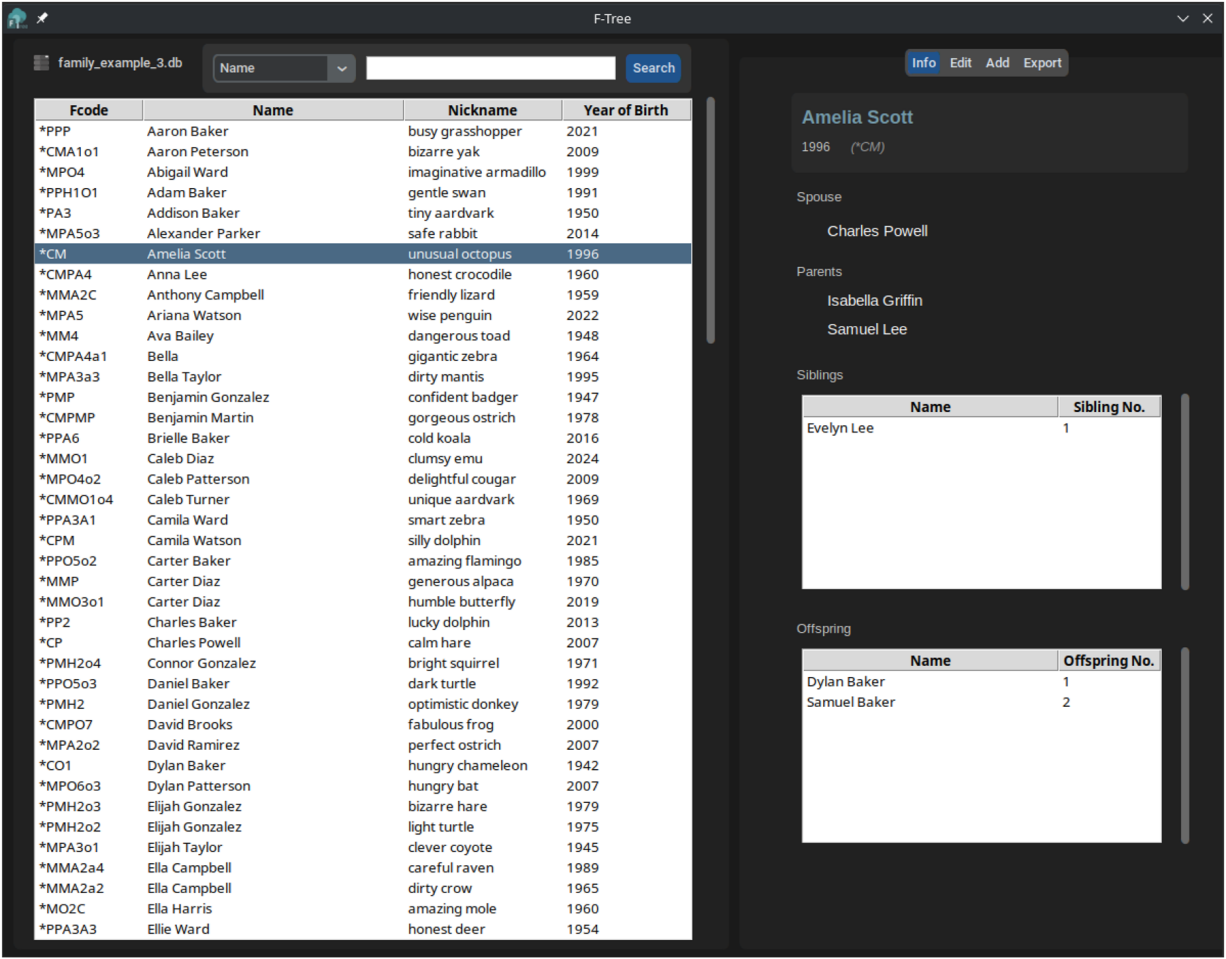
F-Tree main interface loaded with an encoded family. In the right panel is a summary of the direct relationships of the selected Fcode. A tab menu on the upper right allows the management of the database as well as the exportation to several data formats, including the generation of a genealogical tree and a report in PDF. The F-Tree software eases the manipulation of data encoded with the Fcode algorithm.

In order to ease the access to the Fcode encoding method, we developed F-Tree (https://github.com/Dannyzimmer/F-Tree), a graphical user interface for managing and visualizing genealogical data using Fcodes (Figure 2) that relies on Fcode CLI functionalities.

F-Tree offers a set of options to take advance of Fcodes, manage and explore a family. Regarding **encoding and data management**, F-Tree supports the import of families in Fdata format, TSV format (an extended version of Fdata with additional columns), and SQLite format (.db), which is the software’s default working format. It enables the expansion of the family through interactive menus, ensuring no duplicates are created, and also allows for the removal of members. The database can be also exported to TSV. Remarkably, as an open-source software, F-Tree utilizes public domain resources in both its source code and data storage. Efforts have been made to ensure data transparency throughout all stages of the process, aligning the software and data with the FAIR (Findable, Accessible, Interoperable, and Reusable) principles and the FAIR4RS (FAIR for Research Software) guidelines [4]. This guarantees that any data generated with this method will remain accessible and usable, even if F-Tree becomes unavailable, ensuring long-term sustainability and reproducibility.

The main F-Tree panel provides a tabular view of all individuals in the family tree (Figure 2). It includes a search bar that allows users to filter the family members by column using regular expressions. This feature enhances the exploration and visualization of the family tree data. For each selected individual, two specific panels can be used: (i) an “Info” panel that displays the direct relationships of the selected individual; (ii) an “Edit” panel that allows editing such person’s information, including its nickname, date of birth and biography. Finally, in the “Export” panel, both the complete table and the subset generated from a search can be used to create a family tree in PDF format, along with a report detailing the individuals and their relationships within the database.

### 3.3 Estimation of the inbreeding coefficient

**Figure 3:**
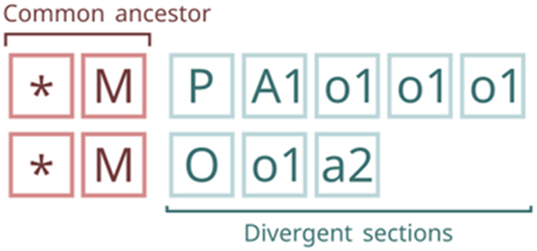
Common ancestor and divergent sections of a pair of Fcodes. The common ancestor corresponds to the last position with matching layers, provided no “spousehood” layer (C) is present in the unmatched layers, which are referred to as the “divergent” section.

As previously mentioned, the relationship between two Fcodes that share the same origin can be traced. This allows for the identification of a common path, which, under certain conditions, may correspond to the common ancestor of both Fcodes (Figure 3).

The common ancestor between two Fcodes is the last position of matching layers between them, provided there is no “spousehood” layer (C) in the divergent section. The divergent section is the one with unmatched layers (Figure 2).

Once the common ancestor is identified, we can use Wright’s inbreeding coefficient equation to estimate the inbreeding coefficient:

$$F={\left(\frac{1}{2}\right)}^{\text{D}{\text{S}}_{1}+\text{D}{\text{S}}_{2}}$$

For DS_1_ and DS_2_ being the depth of each of the divergent sections. For example, given the Fcodes *P3 and *PMO1a1, the common ancestor is *P3, divergent sections are (“MO1a1”, “”), depths of the divergent sections are (3, 0), and the coefficient of inbred is:

$$F={\left(\frac{1}{2}\right)}^{3+0}=0.125$$

Which is the expected inbreed coefficient between first cousins.

### 3.4 LLM models and Fcodes

Currently, NLP processing tasks are addressed by LLMs, capable of generating text on almost any topic, including programming and structured languages, with a great level of performance. In this section, we explore the suitability of using two of the most powerful LLMs, namely ChatGPT-4o and Gemini Advanced 1.5 Pro, for inferring family relationships from different narrative texts and encode them using the Fcodes method.

To do so, we designed an initial prompt that presents a simplified version about how the Fcode algorithm works along with a small test to verify their understanding of the system. Supplementary Material 1 presents the ten different text cases as well as such initial prompt. For each of the ten text cases, the expected Fcodes that the model should construct are presented, along with the responses given by each model. Some text have been invented by the authors while others have been taken from Wikipedia (e.g. Marie Curie or King Arthur’s family), books (e.g. La Regenta or Genesis), or online websites.

Table 2 summarizes the results obtained by both models in the ten text cases. As it can be seen, ChatGPT-4o nailed it in extracting the six relationships, while Gemini Advanced 1.5 Pro made two fails. Regarding the narrative text cases, it is important to note that some of them include an addition or subtraction to the count to indicate that, for instance, in case 3 (Charles Darwin short bio), ChatGPT-4o proposed five additional relationships than the expected ones to represent their siblings. This is interesting and we were not expecting them as their names are not listed and the only reference is that “Charles Darwin is the fifth of six children”. In other cases, as the fifth, probably the hardest one, both models failed in encoding three relationships and even missed one relationship (hence the −1).

**Table 2:** Summary of the results obtained by the LLM models ChatGPT-4o and Gemini Advanced 1.5 Pro across ten text cases. An initial prompt explaining the Fcode algorithm, along with an example, was provided as a starting point. The “Count” column shows the number of Fcodes identified by each model, represented as fractions of the expected Fcodes. The “%” column indicates the proportion of expected Fcodes correctly identified by each model.

	ChatGPT-4o		Gemini Advanced 1.5 Pro	
	Count	%	Count	%
Initial	6/6	100.00 %	4/6	66.67 %
1	4/5	80.00 %	2/5	40.00 %
2	3/5	60.00 %	3/5	60.00 %
3	5/5 + 5^a^	100.00 %	3/5	60.00 %
4	4/4	100.00 %	4/4	100.00 %
5	2/6 − 1^b^	33.33 %	4/6 − 1^b^	66.67 %
6	7/7	100.00 %	7/7	100.00 %
7	8/8	100.00 %	8/8	100.00 %
8	9/9 + 1^c^	100.00 %	9/9	100.00 %
9	6/6	100.00 %	2/6	33.33 %
10	6/6	100.00 %	4/6	66.67 %
Global	89.55 %		83.33 %	

^a^The +5 here means that ChatGPT-4o proposed 5 more encodings than the expected ones. ^b^The −1 here means that both models failed to infer one relationship (*A., Sister of Samuel, mother of Timothy). ^c^The +1 here means that ChatGPT-4o proposed one more relationship than the expected ones.

These results are promising and demonstrate the suitability of combining the Fcodes method with LLMs to infer relationships from free narrative texts, thus structuring the knowledge contained in them. It would be interesting to explore how these models can be integrated into practical workflows, considering challenges such as accuracy in historical or medical data and the impact of linguistic and cultural biases.

## 4 Conclusions

In our previous work, we developed Fcodes, a straightforward algorithm for kinship encoding. The Fcodes encoding method is highly flexible, preserving many properties of family structures, as demonstrated by its ability to estimate the inbreeding coefficient from two isolated Fcodes. To further our goal of making Fcodes a simple and accessible framework for managing kinship data, we introduced F-Tree, a graphical user interface. F-Tree represents a significant improvement in the accessibility of the algorithm, enabling its use in diverse research contexts and offering a powerful, flexible tool for managing genealogical data.

Additionally, we explore the integration of LLMs with Fcodes to automatically infer family relationships from narrative texts. The promising results obtained suggest substantial potential for automating the processing of unstructured data, such as that found in medical records, civil registrations, and historical documents.

Overall, the advancements presented in this work establish Fcodes as a comprehensive solution for encoding and analyzing family relationships, unifying accessibility for non-technical users with robustness for advanced computational analysis. It continues to bridge the gap between traditional and modern methods of representing family structures, with significant potential applications in both social sciences and life sciences.

## Supplementary Material

Supplementary Material Details
