# Supplementary material for: Fcodes update: a kinship encoding framework with F-Tree GUI & LLM inference
Source: J Integr Bioinform. 2025 Mar 31;22(2):20240046. doi: 10.1515/jib-2024-0046 (PMC12569577; doi:10.1515/jib-2024-0046)
Supplement: Supplementary file 1 — Supplementary Material Details [file j_jib-2024-0046_suppl_001.docx]

*This document contains the messages that will be sent to the NLP models ChatGPT-4o and Gemini Advanced 1.5 Pro, in order to evaluate their ability to encode relationships using the Fcodes method. The section “Initial prompt” includes the initial text that will be presented to the models so they can understand how a simplified version of the Fcode algorithm works, along with a small test to verify their understanding of the system. The remaining sections correspond to 10 different texts. At the end of each of these 10 sections, the expected Fcodes that the model should construct are presented, along with the responses given by each model.*

# Initial prompt

## Prompt

We have developed an algorithm called "fcodes" to encode kinship relationships.

This is the encoding legend:

* = origin of coordinates (usually oneself)

C = spouse

O = brother

A = sister

H = sibling (sex unknown)

P = father

M = mother

X = parent (sex unknown)

o = son

a = daughter

h = offspring (sex unknown)

Along with the encoding legend, it is essential to address the following considerations in order to write a valid fcode:

- Consideration 1:

1.1. Siblings (O, A, H) and offspring (o, a, h) must be numbered in order of birth. The numbering does not distinguish between sexes.

1.2 In case of being unknown use “-” to indicate that it is the youngest; “.” to indicate that its number is unknown.

- Consideration 2: Parents (P, M) and partners (C) SHOULD BE NUMBERED in order of birth relative to their siblings. For example: *M2, *P1, *C3, *M2O4, *P1O3, or *CM2P7P4.

The Fcode is constructed by sequentially adding letter-number groups (based on considerations 1 and 2) from left to right. Each newly added group refers to the previous one, forming a path from the origin of coordinates (OC) to the person being encoded. In this manner, the kinship is progressively built from left to right as follows:

- * (Me)

- *P1 (My father, the eldest son of my paternal grandparents)

My father, elder among his siblings = (* + P1) = *P1

(*) me = *

(* + P1) my father = *P1

- *M3 (My mother, the third daughter of my maternal grandparents)

My mother, third in age among her siblings = (* + M3) = *M3

(*) me = *

(* + M3) my mother= *M3

- *M3P. (My maternal grandfather, unknown number)

My mother’s father = (* + M3 + P.) = *M3P.

(*) = me = *

(* + M3) = my mother = *M3 (Numbered because consideration 2.1)

(*M3 + P.) = my mother’s father = *M3P.

- *P1M. (My paternal grandmother, unknown number)

My father’s mother = (* + P1 + M.) = *P1M.

(*) = me = *

(* + P1) = my father = *P1

(*P1 + M.) = my father’s mother (unknown number) = *P1M.

-*M3O2a2 (The second daughter of the second brother of my mother)

my mother’s second brother’s daughter = (* + M3 + O2 + a2) = *M3O2a2

(*) = me = *

(* + M3) = my mother = *M3

(*M3 + O2) = my mother’s second brother = *M3O2

(*MO2 + a2) = my mother’s second brother’s daughter = *M3O2a2

- *M3O2C.O-a1:

My mother’s second brother’s spouse’s younger brother’s elder daughter = (* + M3 + O2 + C + O- + a1) = *M3O2CO-a1

(*) = me = *

(* + M3) = my mother = *M3

(*M3 + O2) = my mother’s second brother; my uncle = *M3O2

(*M3O2 + C.) = my uncle’s spouse (unknown number); my aunt-in-law = *M3O2C.

(*M3O2C. + O-) = my aunt-in-law’s younger brother; my uncle-in-law = *M3O2C.O-

(*M3O2CO- + a1) = my uncle-in-law’s first daughter = *M3O2C.O-a1

Here are some more examples of texts and the encodings they should produce:

- Example: “My mother has two elder siblings, Lisa and Kazy. Kazy was the most spoiled girl for being the first. Using me as the origin of coordinates, write the Fcodes of the people in this story.” Encodings:
  - Narrator: (*)
  - Narrator’s mother: (* + M3) = *M3
  - Lisa, narrator’s mother second elder sibling: (*M3 + A2) = *M3A2
  - Kazy, narrator’s mother elder sibling: (*M3 + A1) = *M3A1.
- Example: “When I walked into the room, they started singing "Happy Birthday." It was my fifteenth birthday. There were my parents, Amanda and Leo, and my two little siblings: Sam, bouncing up and down while singing, eager for the cake to arrive, and Tina, wide-eyed as she watched the scene, sucking on her pacifier with delight. Using Amanda as the origin of coordinates, write the Fcodes of the people in this story.”.

Encodings:

- - Amanda: *
  - Leo, Amanda’s spouse (unknown number): (* + C.) = *C.
  - Narrator, Amanda’s elder descendant (the text does not specify sex): (* + h1) = *h1
  - Sam, Amanda’s second son: (* + o2) = *o2
  - Tina, Amanda’s younger daughter, the third in age: (* + a3) = *a3

Taking these considerations into account, produce the encodings for the following text between quotes and justify your choices. Use John as the origin of coordinates and produce as many relationships as necessary:

"John is married to Linda. Both of them are researchers at the University of Columbia. John's father, Ben, is an ex professional football player. John’s mother is also retired and was a secondary school teacher. John has two siblings: Michael, the older one, and Michele, the younger. He is the middle aged sibling.”

## Expected result

John *

Linda C

Ben *P.

John’s mother *M

Michael *O1

Michele *A3

## ChatGPT-4o (6/6)

Using **John** as the origin of coordinates (denoted as *), let’s break down the relationships in the story step by step and encode them using the "fcodes" rules.

### **Relationships and Justifications:**

1. **John** (origin of coordinates) is denoted as *.
   - **John's fcode**: *.
2. **John's wife, Linda**.
   - Linda is John’s spouse. Since there is no information about Linda having other spouses or her order among them, we use the unknown numbering C..
   - **Linda’s fcode**: *C.
3. **John’s father, Ben**.
   - Ben is John’s father. There’s no information regarding Ben’s order among his siblings (if he has any), so we denote his number as unknown P..
   - **Ben’s fcode**: *P.
4. **John’s mother**.
   - John’s mother is mentioned, but her name is not specified. Since she is John’s mother and no sibling order is provided, her number is also unknown (M.).
   - **John’s mother’s fcode**: *M.
5. **John’s older brother, Michael**.
   - Michael is John’s elder sibling (he is the first among the siblings). Therefore, we use the sibling code O with the number 1 because he is the first-born.
   - **Michael’s fcode**: *O1
6. **John’s younger sister, Michele**.
   - Michele is John’s younger sibling, the third-born (since John is the middle child, and there are three siblings). Therefore, she is the third sibling, and we use the sibling code A with the number 3 (indicating she is the third sibling and female).
   - **Michele’s fcode**: *A3

### **Final Encodings:**

- **John**: *
- **Linda (John's wife)**: *C.
- **Ben (John's father)**: *P.
- **John's mother**: *M.
- **Michael (John's elder brother)**: *O1
- **Michele (John's younger sister)**: *A3

### **Justifications:**

- **John**: He is the starting point, so we use *.
- **Linda**: As John’s spouse, her code is C., and since her sibling order (if any) is unknown, we use ..
- **Ben**: Ben is John’s father, so we use P., with the . to indicate his sibling order is unknown.
- **John’s mother**: As John’s mother, we use M., and again, her sibling order is unknown, so we use ..
- **Michael**: Michael is the eldest sibling of John, hence he is assigned O1.
- **Michele**: Michele is the youngest sibling, and as John’s sister, her code is A3 (indicating the third sibling and female).

## Gemini Advanced 1.5 Pro (4/6)

**John:** *

**Linda:** *C (John's spouse, no information on birth order relative to siblings)

**Ben (John's father):** *P1 (Assuming Ben is John's father and no mention of other fathers)

**John's Mother:** *M (No information on birth order relative to siblings)

**Michael:** *O1 (John's older brother)

**Michele:** *A2 (John's younger sister)

# Prompt 1 (invented)

## Prompt

The garden writhed vigorously under the onslaught of autumn winds. On the other side, Rose remained silently for hours, watching from behind the window as the trees danced to the rhythm of a strange melody she could hear through the chimney and beneath the door.

At exactly six o'clock, she would hear her grandmother Miranda, the mother of Alcina, entering the kitchen to prepare dinner. However, her blue eyes remained fixed, focused on the dancing creatures outside. Inside, she was anxious about whether her parents and her sister, out in that gale, would suffer similar contortions.

Karl, her father, left very early each morning to open his practice; shortly followed by Alcina, her mother, and Daniela, her older sister. By the time she woke up, only Miranda was there.

Using Rose as the origin of coordinates, write the Fcodes of the people in this story.

## Expected result

* Rose

*P. Karl

*M. Alcina

*A1 Daniela

*M.M. Miranda

## ChatGPT-4o (4/5)

Using **Rose** as the origin of coordinates (*), let's break down the relationships step by step and apply the rules for encoding kinship using fcodes:

### **Relationships and Justifications:**

1. **Rose** (origin of coordinates) is denoted as *.
   - **Rose’s fcode**: *.
2. **Miranda (Rose’s grandmother, mother of Alcina)**.
   - Miranda is Rose’s maternal grandmother, as she is the mother of Alcina, Rose’s mother. Since Miranda’s sibling order is unknown, we use the code for mother (M.), and for Miranda’s parent relationship, we include that she is Rose’s mother’s parent (*M.P.).
   - **Miranda’s fcode**: *M.P.
3. **Alcina (Rose’s mother)**.
   - Alcina is Rose’s mother. Since Alcina’s sibling order is not provided, we code her simply as Rose’s mother (M.).
   - **Alcina’s fcode**: *M.
4. **Karl (Rose’s father)**.
   - Karl is Rose’s father. Since no sibling order is given for Karl, we use the code for father (P.).
   - **Karl’s fcode**: *P.
5. **Daniela (Rose’s older sister)**.
   - Daniela is Rose’s elder sister, meaning she is the first sibling (since there are only two sisters mentioned). Therefore, we use the code for sister (A) and number her as 1 because she is the first-born sibling.
   - **Daniela’s fcode**: *A1

### **Final Encodings:**

- **Rose**: *
- **Miranda (Rose’s maternal grandmother)**: *M.P.
- **Alcina (Rose’s mother)**: *M.
- **Karl (Rose’s father)**: *P.
- **Daniela (Rose’s elder sister)**: *A1

### **Justifications:**

- **Rose**: She is the origin of coordinates, so her fcode is *.
- **Miranda**: As Rose’s maternal grandmother, we trace the relationship through her mother Alcina, making Miranda encoded as *M.P. (mother of Rose’s mother).
- **Alcina**: Alcina is Rose’s mother, and since her sibling order is not given, we encode her as *M..
- **Karl**: Karl is Rose’s father, encoded as *P..
- **Daniela**: Daniela is Rose’s elder sister, so we use the sister code A with the number 1 (*A1).

[PROMPT]: The correct Fcodes are:

Rose *P.

Karl *M.

Alcina *A1

Daniela *M.M.

Miranda *M.P. is not correct for encoding Miranda, as *M.P. would be Rose's mother's father (the maternal grandfather of Rose). The correct encoding for Miranda is "*M.M.".

You're absolutely right! I made a mistake in encoding Miranda’s relationship. Miranda, as Rose’s **maternal grandmother**, should indeed be encoded as *M.M. rather than *M.P.. Here's the corrected set of Fcodes for the relationships in the story:

### **Corrected Fcodes:**

1. **Rose** (origin of coordinates): *
2. **Karl (Rose’s father)**: *P.
   - P. is used for Rose’s father with unknown sibling order.
3. **Alcina (Rose’s mother)**: *M.
   - M. is used for Rose’s mother, with no information about her sibling order.
4. **Daniela (Rose’s elder sister)**: *A1
   - A1 indicates Daniela is the elder sister (first sibling).
5. **Miranda (Rose’s maternal grandmother, Alcina’s mother)**: *M.M.
   - M.M. correctly represents Miranda as the mother of Rose’s mother Alcina, making her Rose’s maternal grandmother.

### **Justification for Miranda:**

- Miranda is **Alcina’s mother**, and therefore Rose’s **maternal grandmother**. The correct encoding for this relationship is *M.M. because we are tracing the lineage from Rose to her mother and then to her mother’s mother (Rose's maternal grandmother).

Thank you for pointing that out!

## Gemini Advanced 1.5 Pro (2/5)

**Rose:** *

**Miranda (Rose's grandmother):** *M2P. (We know Miranda is Alcina's mother, making her Rose's maternal grandmother. We don't know her birth order among her own siblings, hence "M." with no number. We also don't know her husband's (Rose's maternal grandfather's) birth order, hence "P." with no number.)

**Alcina (Rose's mother):** *M2 (Alcina is Rose's mother, and we know she has at least one sibling, Miranda. We don't know Alcina's birth order among her siblings, so we use "2" arbitrarily.)

**Karl (Rose's father):** *C. (Karl is Rose's father through her mother, Alcina. We don't know his birth order among his siblings, hence "C." with no number.)

**Daniela (Rose's sister):** *A1 (Daniela is Rose's older sister.)

# Prompt 2 (invented)

## Prompt

Ben was hurrying up the avenue, having just dropped off his son Ken at daycare, when his wife Natalia called to tell him that his father-in-law was at the house. They had completely forgotten about his arrival in town! After becoming a widower, Bertolt had decided to leave the village and buy an apartment near where his only and beloved daughter lived.

By the time Ben finally got home, the widower was absentmindedly flipping through a dry cleaner's leaflet while Jim, their other son, played the guitar.

–Dad –said Natalia–; Ben just got here. Shall we go for a walk?

Using Bertolt as the origin of coordinates, write the Fcodes of the people in this story.

## Expected result

* Bertolt

*a1 Natalia

*a1C. Ben

*a1o1 Jim

*a1o2 Ken

## ChatGPT-4o (3/5)

Using **Bertolt** as the origin of coordinates (denoted as *), we can break down the relationships from Bertolt's perspective and generate the correct Fcodes for each person in the story, adhering to the encoding rules.

### **Relationships and Justifications:**

1. **Bertolt** (origin of coordinates) is denoted as *.
   - **Bertolt’s fcode**: *.
2. **Natalia (Bertolt’s daughter)**.
   - Natalia is Bertolt’s only child, meaning she is his daughter, and she is the first and only child. Therefore, we encode her as Bertolt's daughter using a1.
   - **Natalia’s fcode**: *a1.
3. **Ben (Natalia’s husband, Bertolt’s son-in-law)**.
   - Ben is Natalia’s husband, making him Bertolt’s son-in-law. We use the spouse code C since Ben is the spouse of Bertolt’s daughter (Natalia), and his sibling order is unknown (C.).
   - **Ben’s fcode**: *a1C.
4. **Ken (Bertolt’s grandson, Ben and Natalia’s son)**.
   - Ken is Bertolt’s grandson, the son of Natalia and Ben. Since the birth order is not provided, we assume Ken is the elder son (since Jim is introduced later) and use the code o1 for the first son.
   - **Ken’s fcode**: *a1o1
5. **Jim (Bertolt’s younger grandson)**.
   - Jim is Bertolt’s younger grandson, the second son of Natalia and Ben. Therefore, we use the code o2 for the second son.
   - **Jim’s fcode**: *a1o2

### **Final Fcodes:**

- **Bertolt**: *
- **Natalia (Bertolt’s daughter)**: *a1
- **Ben (Bertolt’s son-in-law)**: *a1C.
- **Ken (Bertolt’s elder grandson)**: *a1o1
- **Jim (Bertolt’s younger grandson)**: *a1o2

### **Justifications:**

- **Bertolt**: As the origin of coordinates, his fcode is *.
- **Natalia**: As Bertolt’s only and beloved daughter, she is encoded as *a1 (first daughter).
- **Ben**: As Natalia’s husband and Bertolt’s son-in-law, Ben is encoded as *a1C. (spouse of the first daughter, with unknown sibling order).
- **Ken**: Ken is the elder son of Bertolt’s daughter, so we use *a1o1 (first son).
- **Jim**: Jim, the younger son, is encoded as *a1o2 (second son).

## Gemini Advanced 1.5 Pro (3/5)

Okay, let's give this another try, with Bertolt as the origin of coordinates:

- **Bertolt:** *
- **Natalia (Bertolt's daughter):** *a1 (His only daughter)
- **Ben (Natalia's husband):** *a1C. (Unknown if Ben has siblings)
- **Ken (Natalia and Ben's son):** *a1o1 (Their first-mentioned son)
- **Jim (Natalia and Ben's other son):** *a1o2 (Mentioned after Ken)

# Prompt 3 - Charles Darwin (Wikipedia)

## Prompt

Darwin was born in Shrewsbury, Shropshire, on 12 February 1809, at his family's home, The Mount. He was the fifth of six children of wealthy society doctor and financier Robert Darwin and Susannah Darwin (née Wedgwood). His grandfathers Erasmus Darwin (father’s side) and Josiah Wedgwood (mother’s side) were both prominent abolitionists. Erasmus Darwin had praised general concepts of evolution and common descent in his Zoonomia (1794), a poetic fantasy of gradual creation including undeveloped ideas anticipating concepts his grandson expanded.

Using Charles Darwing as the origin of coordinates, write the Fcodes of the people in this story.

## Expected result

* Charles Darwing

*P. Robert Darwin

*M. Susannah Darwin

*P.P. Erasmus Darwin

*M.P. Josiah Wedgwood

## ChatGPT-4o (5/5) + 5

Using **Charles Darwin** as the origin of coordinates (denoted as *), let's break down the relationships step by step and generate the correct Fcodes for each person in the story.

### **Relationships and Justifications:**

1. **Charles Darwin** (origin of coordinates) is denoted as *.
   - **Charles Darwin’s fcode**: *.
2. **Robert Darwin (Charles's father)**.
   - Robert is Charles Darwin’s father. Since no information is provided about Robert’s sibling order, we encode him as P..
   - **Robert Darwin’s fcode**: *P.
3. **Susannah Darwin (née Wedgwood, Charles's mother)**.
   - Susannah is Charles Darwin’s mother. No sibling order is provided for Susannah, so we encode her as M..
   - **Susannah Darwin’s fcode**: *M.
4. **Erasmus Darwin (Charles’s paternal grandfather)**.
   - Erasmus Darwin is Charles Darwin’s paternal grandfather (Robert’s father). Therefore, we trace this relationship through Robert, encoding Erasmus as Charles’s father’s father, or P.P..
   - **Erasmus Darwin’s fcode**: *P.P.
5. **Josiah Wedgwood (Charles’s maternal grandfather)**.
   - Josiah Wedgwood is Charles Darwin’s maternal grandfather (Susannah’s father). Therefore, we trace the relationship through Susannah and encode him as Charles’s mother’s father, or M.P..
   - **Josiah Wedgwood’s fcode**: *M.P.
6. **Charles’s siblings**.
   - Charles Darwin is the fifth of six children. His siblings must be numbered, but they are not explicitly named in the text, so we could encode them as siblings using O for brothers, A for sisters, or H for siblings if their sex is unknown, and number them according to their birth order. For simplicity, let’s assume there is at least one brother and one sister.
   - **Charles’s eldest sibling** (unknown gender): *H1
   - **Charles’s second sibling** (unknown gender): *H2
   - **Charles’s third sibling** (unknown gender): *H3
   - **Charles’s fourth sibling** (unknown gender): *H4
   - **Charles’s younger sibling** (sixth child, unknown gender): *H6

### **Final Fcodes:**

- **Charles Darwin**: *
- **Robert Darwin (Charles’s father)**: *P.
- **Susannah Darwin (Charles’s mother)**: *M.
- **Erasmus Darwin (Charles’s paternal grandfather)**: *P.P.
- **Josiah Wedgwood (Charles’s maternal grandfather)**: *M.P.
- **Charles’s eldest sibling**: *H1
- **Charles’s second sibling**: *H2
- **Charles’s third sibling**: *H3
- **Charles’s fourth sibling**: *H4
- **Charles’s younger sibling (sixth)**: *H6

### **Justifications:**

- **Charles Darwin**: As the origin of coordinates, Charles is denoted as *.
- **Robert Darwin**: Robert is Charles’s father, encoded as *P. since his sibling order is unknown.
- **Susannah Darwin**: Susannah is Charles’s mother, encoded as *M. since her sibling order is unknown.
- **Erasmus Darwin**: Erasmus is Charles’s paternal grandfather, encoded as *P.P. (father’s father).
- **Josiah Wedgwood**: Josiah is Charles’s maternal grandfather, encoded as *M.P. (mother’s father).
- **Charles’s siblings**: Charles is the fifth of six children, so his siblings are encoded using sibling codes H, with numbers representing their birth order. Their genders are unspecified in the text.

## Gemini Advanced 1.5 Pro (3/5)

You're asking for the fcodes of people related to Charles Darwin, with Charles as the origin of coordinates. Here's the breakdown:

- **Charles Darwin:** *
- **Robert Darwin (Charles' father):** *P1 (We can assume he's *P1 as there's no mention of other fathers)
- **Susannah Darwin (née Wedgwood) (Charles' mother):** *M1 (Same assumption as above)
- **Erasmus Darwin (Charles' paternal grandfather):** *P1P. (Father of Robert, unknown birth order)
- **Josiah Wedgwood (Charles' maternal grandfather):** *M1P. (Father of Susannah, unknown birth order)

[PROMPT]: The codes are almost correct, but when we have no information about birth number, we use a dot "." instead of a number. The correct answer is:

* Charles Darwing

*P. Robert Darwin

You're absolutely correct! I apologize for the mistake. I am still under development and learning to be more accurate.

Thanks for pointing out the correction and helping me learn. I'll strive to be more precise in the future.

Here's the fully corrected list, using "." for unknown birth order:

- **Charles Darwin:** *
- **Robert Darwin:** *P.
- **Susannah Darwin:** *M.
- **Erasmus Darwin:** *P.P.
- **Josiah Wedgwood:** *M.P.

# Prompt 4 - soulveda.com

## Prompt

James found himself nestled in the cozy corner of his living room, enjoying a cup of evening tea, when his phone alerted him of an incoming video call. A warm smile graced James’s face as he answered the call and one by one, the faces of his siblings came up on the screen, all from different corners of the world.

Lena, the family’s eldest, had settled in Norway and had a thriving career in finance. Tom, the middle one, and Emily, the youngest, beamed from their university campus in Melbourne.

As they chatted and caught up on each other’s lives, James felt a deep sense of nostalgia. He missed the days when they all lived under the same roof, sharing laughter and stories around the dinner table. A thought began to form in his mind almost on an impulse — a plan to bring them all together once again.

“Hey, I’ve been thinking,” James began, his voice brimming with a hint of excitement, “How about a family reunion?”

Using James as the origin of coordinates, write the Fcodes of the people in this story.

## Expected result

* James

*A1 Lena

*O2 Tom

*A3 Emily

## ChatGPT-4o (4/4)

Using **James** as the origin of coordinates (denoted as *), let’s break down the relationships and generate the correct Fcodes for each person in the story, following the encoding rules:

### **Relationships and Justifications:**

1. **James** (origin of coordinates).
   - James is the focal point, so he is represented by *.
   - **James’s fcode**: *.
2. **Lena (James’s eldest sibling)**.
   - Lena is James’s eldest sibling. Since she is the first sibling, we use A1 for the first sister.
   - **Lena’s fcode**: *A1.
3. **Tom (James’s middle sibling)**.
   - Tom is James’s brother, the middle sibling. Therefore, we use O2 for the second brother.
   - **Tom’s fcode**: *O2.
4. **Emily (James’s youngest sibling)**.
   - Emily is James’s younger sister, the third sibling and the youngest. Therefore, we use A3 for the third sister.
   - **Emily’s fcode**: *A3.

### **Final Fcodes:**

- **James**: *
- **Lena (James’s eldest sister)**: *A1
- **Tom (James’s middle brother)**: *O2
- **Emily (James’s youngest sister)**: *A3

### **Justifications:**

- **James**: As the origin of coordinates, James is represented by *.
- **Lena**: Since Lena is James’s eldest sister, her fcode is *A1 (first sister).
- **Tom**: Tom is James’s middle sibling and his brother, so his fcode is *O2 (second brother).
- **Emily**: Emily is James’s youngest sister, so her fcode is *A3 (third sister).

This encoding accurately captures the sibling relationships from James’s perspective.

## Gemini Advanced 1.5 Pro (4/4)

**James:** *

**Lena:** *A1 (Eldest sibling)

**Tom:** *O2 (Middle sibling, and we can infer he's male from the name)

**Emily:** *A3 (Youngest sibling)

# Prompt 5 (invented)

## Prompt

Jasmine, the grandmother, Mathiew, the father, and Timothy, the son, were all captivated by the beautiful music drifting through the window. There was no doubt about it—Sarah was a true virtuoso, one of those rare souls that come to dazzle the world once every many years.

Samuel, the father of Sarah, watched the trio proudly from the window, savoring the genuine look of surprise on his brother-in-law's face. Though Timothy was his sister's son, it was clear he hadn’t inherited the gift of music—or rather, he had squandered it by not indulging in what Samuel considered “quality music.”

Using Samuel as the origin of coordinates, write the Fcodes of the people in this story.

## Expected result

* Samuel

*a. Sarah

*A. Sister of Samuel, mother of Timothy

*A.o. Timothy

*A.C.M. Jasmine

*A.C. Mathiew

## ChatGPT-4o (2/6) - 1

Using **Samuel** as the origin of coordinates (denoted as *), we will break down the relationships and generate the correct Fcodes for each person in the story based on their relationship to Samuel.

### **Relationships and Justifications:**

1. **Samuel** (origin of coordinates).
   - Samuel is the focal point, so he is represented by *.
   - **Samuel’s fcode**: *.
2. **Sarah (Samuel’s daughter)**.
   - Sarah is Samuel’s daughter. Since no other offspring are mentioned, we encode Sarah as Samuel's first daughter using a1.
   - **Sarah’s fcode**: *a1.
3. **Jasmine (Samuel’s mother-in-law)**.
   - Jasmine is the **mother of Samuel’s wife**, making her Samuel’s mother-in-law. Therefore, we trace the relationship through Samuel’s wife (whose sibling order is unknown), using C.M. to represent Samuel’s spouse’s mother.
   - **Jasmine’s fcode**: *C.M.
4. **Mathiew (Samuel’s brother-in-law, Timothy’s father)**.
   - Mathiew is Samuel’s brother-in-law, meaning he is Samuel’s spouse’s brother. We encode him as C.O. to represent Samuel’s wife’s brother (since his sibling order is not provided).
   - **Mathiew’s fcode**: *C.O.
5. **Timothy (Samuel’s nephew, Mathiew’s son)**.
   - Timothy is Mathiew’s son, and thus Samuel’s nephew. To encode him, we trace through Mathiew (C.O.) and then encode Timothy as Mathiew’s son using o1 (as there’s no indication that Mathiew has other children).
   - **Timothy’s fcode**: *C.O.o1

### **Final Fcodes:**

- **Samuel**: *
- **Sarah (Samuel’s daughter)**: *a1
- **Jasmine (Samuel’s mother-in-law)**: *C.M.
- **Mathiew (Samuel’s brother-in-law)**: *C.O.
- **Timothy (Samuel’s nephew)**: *C.O.o1

### **Justifications:**

- **Samuel**: As the origin of coordinates, Samuel is represented by *.
- **Sarah**: Sarah is Samuel’s daughter, so her fcode is *a1 (first daughter).
- **Jasmine**: Jasmine is Samuel’s mother-in-law (his spouse’s mother), so her fcode is *C.M..
- **Mathiew**: Mathiew is Samuel’s brother-in-law (his spouse’s brother), so his fcode is *C.O..
- **Timothy**: Timothy is Mathiew’s son, so his fcode is *C.O.o1 (first son of Mathiew).

## Gemini Advanced 1.5 Pro (4/6) - 1

**Samuel:** *

**Jasmine (Samuel's sister's mother-in-law):** *A.M. (We don't know if Samuel has siblings, so we use "A." to represent his sister with an unknown birth order. Then, "M." represents Jasmine as the mother of Samuel's sister's husband, with an unknown birth order.)

**Mathieu (Samuel's sister's husband):** *A.C. (Similar to above, "A." for Samuel's sister with unknown birth order, and "C." for her husband with an unknown birth order.)

**Timothy (Samuel's sister's son):** *A.o1 (We can assume Timothy is the first son, as no other children are mentioned before him.)

**Sarah (Samuel's niece):** *A.a1 (We can assume Sarah is the first daughter, as no other children are mentioned before her.)

# Prompt 6 - Marie Curie (Wikipedia)

## Prompt

Maria Skłodowska was born in Warsaw, in Congress Poland in the Russian Empire, on 7 November 1867, the fifth and youngest child of well-known teachers Bronisława, née Boguska, and Władysław Skłodowski. The elder siblings of Maria (nicknamed Mania) were Zofia (born 1862, nicknamed Zosia), Józef (born 1863, nicknamed Józio), Bronisława (born 1865, nicknamed Bronia) and Helena (born 1866, nicknamed Hela).

Using Maria Skłodowska as the origin of coordinates, write the Fcodes of her family.

## Expected result

* Maria Skłodowska

*M. Bronisława Skłodowska

*P. Władysław Skłodowski

*A1 Zofia (Zosia)

*O2 Józef (Józio)

*A3 Bronisława (Bronia)

*A4 Helena (Hela)

## ChatGPT-4o (7/7)

Using **Maria Skłodowska** as the origin of coordinates (denoted as *), let’s break down the relationships and generate the correct Fcodes for each family member based on the birth order and roles described in the story.

### **Relationships and Justifications:**

1. **Maria Skłodowska (origin of coordinates)**.
   - Maria is the focal point, so her Fcode is *.
   - **Maria’s fcode**: *.
2. **Władysław Skłodowski (Maria’s father)**.
   - Władysław is Maria’s father. No sibling order is provided, so we use P. to denote the father with an unknown birth order.
   - **Władysław’s fcode**: *P.
3. **Bronisława Boguska (Maria’s mother)**.
   - Bronisława is Maria’s mother. Similarly, no sibling order is provided, so we use M. for the mother with an unknown birth order.
   - **Bronisława’s fcode**: *M.
4. **Zofia (Zosia) (Maria’s eldest sister)**.
   - Zofia is Maria’s eldest sibling and a sister. Therefore, she is encoded as A1 (first sister).
   - **Zofia’s fcode**: *A1.
5. **Józef (Józio) (Maria’s eldest brother)**.
   - Józef is Maria’s second sibling and first brother. Therefore, he is encoded as O2 (second sibling, first brother).
   - **Józef’s fcode**: *O2.
6. **Bronisława (Bronia) (Maria’s second sister)**.
   - Bronisława (Bronia) is Maria’s third sibling and second sister. Therefore, she is encoded as A3 (third sibling, second sister).
   - **Bronisława’s fcode**: *A3.
7. **Helena (Hela) (Maria’s third sister)**.
   - Helena is Maria’s fourth sibling and third sister. Therefore, she is encoded as A4 (fourth sibling, third sister).
   - **Helena’s fcode**: *A4.

### **Final Fcodes:**

- **Maria Skłodowska**: *
- **Władysław Skłodowski (Maria’s father)**: *P.
- **Bronisława Boguska (Maria’s mother)**: *M.
- **Zofia (Zosia) (Maria’s eldest sister)**: *A1
- **Józef (Józio) (Maria’s eldest brother)**: *O2
- **Bronisława (Bronia) (Maria’s second sister)**: *A3
- **Helena (Hela) (Maria’s third sister)**: *A4

### **Justifications:**

- **Maria**: As the origin of coordinates, Maria is represented by *.
- **Władysław**: Maria’s father, encoded as *P. since no sibling order is provided.
- **Bronisława (mother)**: Encoded as *M. since her sibling order is unknown.
- **Zofia (Zosia)**: Eldest sister, encoded as *A1.
- **Józef (Józio)**: Eldest brother, encoded as *O2 (second sibling, first brother).
- **Bronisława (Bronia)**: Second sister, encoded as *A3.
- **Helena (Hela)**: Third sister, encoded as *A4.

This set of Fcodes represents Maria Skłodowska’s family relationships from her perspective.

## Gemini Advanced 1.5 Pro (7/7)

**Maria Skłodowska:** *

**Bronisława Skłodowski (née Boguska):** *M. (Maria's mother, birth order unknown)

**Władysław Skłodowski:** *P. (Maria's father, birth order unknown)

**Zofia Skłodowska:** *A1 (Maria's oldest sibling)

**Józef Skłodowski:** *O2 (Maria's older brother, second oldest sibling)

**Bronisława Skłodowska:** *A3 (Maria's older sister, third oldest sibling)

**Helena Skłodowska:** *A4 (Maria's older sister, fourth oldest sibling)

# Prompt 7 - King Arthur's family (Wikipedia)

## Prompt

The genealogies from the 13th-century Mostyn MS. 117 assert that Arthur is the son of Uthyr, the son of Custennin, the son of Cynfawr, the son of Tudwal, the son of Morfawr [....]

Regarding Arthur's own family, his wife is consistently stated to be Gwenhwyfar, usually the daughter of King Ogrfan Gawr.

Using King Arthur as the origin of coordinates, write the Fcodes of his family.

## Expected result

* King Arthur

*C. Gwenhwyfar

*C.P. King Ogrfan Gawr

*P. Uthyr

*P.P. Custennin

*P.P.P. Cynfawr

*P.P.P.P. Tudwal

*P.P.P.P.P. Morfawr

## ChatGPT-4o (8/8)

Using **King Arthur** as the origin of coordinates (denoted as *), let's break down his family relationships and generate the appropriate Fcodes based on the genealogical information provided.

### **Relationships and Justifications:**

1. **King Arthur (origin of coordinates)**.
   - Arthur is the focal point, so his Fcode is *.
   - **Arthur’s fcode**: *.
2. **Uthyr (Arthur’s father)**.
   - Uthyr is Arthur's father, commonly known as Uther Pendragon. Since no sibling order is provided, we encode him as P..
   - **Uthyr’s fcode**: *P.
3. **Custennin (Arthur’s paternal grandfather)**.
   - Custennin is Arthur’s paternal grandfather (Uthyr’s father). Therefore, Custennin is encoded as Arthur’s father’s father, P.P..
   - **Custennin’s fcode**: *P.P.
4. **Cynfawr (Arthur’s paternal great-grandfather)**.
   - Cynfawr is Arthur’s great-grandfather (Custennin’s father). Therefore, Cynfawr is encoded as Arthur’s father’s father’s father, P.P.P..
   - **Cynfawr’s fcode**: *P.P.P.
5. **Tudwal (Arthur’s paternal great-great-grandfather)**.
   - Tudwal is Arthur’s great-great-grandfather (Cynfawr’s father). Tudwal is encoded as P.P.P.P..
   - **Tudwal’s fcode**: *P.P.P.P.
6. **Morfawr (Arthur’s paternal great-great-great-grandfather)**.
   - Morfawr is Arthur’s great-great-great-grandfather (Tudwal’s father). He is encoded as P.P.P.P.P..
   - **Morfawr’s fcode**: *P.P.P.P.P.
7. **Gwenhwyfar (Guinevere) (Arthur’s wife)**.
   - Gwenhwyfar (Guinevere) is consistently noted as Arthur’s wife. Since no sibling order is provided for Gwenhwyfar, she is encoded as C. (spouse).
   - **Gwenhwyfar’s fcode**: *C.
8. **Ogrfan Gawr (Gwenhwyfar’s father)**.
   - Ogrfan Gawr is Gwenhwyfar’s father (Arthur’s father-in-law). Therefore, Ogrfan is encoded as Arthur’s wife’s father, C.P..
   - **Ogrfan Gawr’s fcode**: *C.P.

### **Final Fcodes:**

- **King Arthur**: *
- **Uthyr (Arthur’s father)**: *P.
- **Custennin (Arthur’s paternal grandfather)**: *P.P.
- **Cynfawr (Arthur’s paternal great-grandfather)**: *P.P.P.
- **Tudwal (Arthur’s paternal great-great-grandfather)**: *P.P.P.P.
- **Morfawr (Arthur’s paternal great-great-great-grandfather)**: *P.P.P.P.P.
- **Gwenhwyfar (Arthur’s wife)**: *C.
- **Ogrfan Gawr (Gwenhwyfar’s father)**: *C.P.

### **Justifications:**

- **Arthur**: As the origin of coordinates, Arthur is represented by *.
- **Uthyr**: Arthur’s father, encoded as *P. since no sibling order is provided.
- **Custennin**: Arthur’s paternal grandfather, encoded as *P.P..
- **Cynfawr**: Arthur’s paternal great-grandfather, encoded as *P.P.P..
- **Tudwal**: Arthur’s paternal great-great-grandfather, encoded as *P.P.P.P..
- **Morfawr**: Arthur’s paternal great-great-great-grandfather, encoded as *P.P.P.P.P..
- **Gwenhwyfar**: Arthur’s wife, encoded as *C..
- **Ogrfan Gawr**: Arthur’s father-in-law, encoded as *C.P..

This set of Fcodes represents King Arthur's family relationships according to the genealogies provided.

## Gemini Advanced 1.5 Pro (8/8)

**King Arthur:** *

**Gwenhwyfar (Arthur's wife):** *C. (No information on her birth order among siblings)

**Uthyr Pendragon (Arthur's father):** *P. (No information on his birth order)

**Custennin (Arthur's grandfather):** *P.P.

**Cynfawr (Arthur's great-grandfather):** *P.P.P.

**Tudwal (Arthur's great-great-grandfather):** *P.P.P.P.

**Morfawr (Arthur's great-great-great-grandfather):** *P.P.P.P.P.

**Ogrfan Gawr (Gwenhwyfar's father):** *C.P. (No information on his birth order)

# Prompt 8 - Genesis Chapter 4

## Prompt

1 Adam had intercourse with his wife Eve, and she conceived and gave birth to Cain. 'I have acquired a man with the help of Yahweh,' she said.

2 She gave birth to a second child, Abel, the brother of Cain.

[...]

17 Cain had intercourse with his wife, and she conceived and gave birth to Enoch. He became the founder of a city and gave the city the name of his son Enoch.

18 Enoch fathered Irad, and Irad fathered Mehujael; Mehujael fathered Methushael, and Methushael fathered Lamech.

Using Adam as the origin of coordinates, write the Fcodes of his family.

## Expected result

* Adam

*C. Eve

*o1 Cain

*o2 Abel

*o1o1 Enoch

*o1o1o1 Irad

*o1o1o1o1 Mehujael

*o1o1o1o1o1 Methushael

*o1o1o1o1o1o1 Lamech

## ChatGPT-4o (9/9) + 1

Using **Adam** as the origin of coordinates (denoted as *), let's break down his family relationships and generate the appropriate Fcodes based on the genealogical details provided in the story.

### **Relationships and Justifications:**

1. **Adam (origin of coordinates)**.
   - Adam is the focal point, so his Fcode is *.
   - **Adam’s fcode**: *.
2. **Eve (Adam’s wife)**.
   - Eve is Adam’s wife. Since no sibling order is provided for Eve, we encode her as C. (spouse).
   - **Eve’s fcode**: *C.
3. **Cain (Adam’s eldest son)**.
   - Cain is Adam’s firstborn son. Therefore, he is encoded as o1 (first son).
   - **Cain’s fcode**: *o1.
4. **Abel (Adam’s second son)**.
   - Abel is Adam’s second son, so he is encoded as o2 (second son).
   - **Abel’s fcode**: *o2.
5. **Cain’s wife**.
   - Cain’s wife is not named, but she is Cain’s spouse. Since no sibling order is provided for her, she is encoded as C. (spouse of Cain).
   - **Cain’s wife’s fcode**: *o1C.
6. **Enoch (Cain’s son)**.
   - Enoch is Cain’s first son, so he is encoded as *o1o1 (Cain’s first son).
   - **Enoch’s fcode**: *o1o1.
7. **Irad (Enoch’s son)**.
   - Irad is Enoch’s son. He is encoded as *o1o1o1 (Enoch’s first son).
   - **Irad’s fcode**: *o1o1o1.
8. **Mehujael (Irad’s son)**.
   - Mehujael is Irad’s son, encoded as *o1o1o1o1 (Irad’s first son).
   - **Mehujael’s fcode**: *o1o1o1o1.
9. **Methushael (Mehujael’s son)**.
   - Methushael is Mehujael’s son, encoded as *o1o1o1o1o1 (Mehujael’s first son).
   - **Methushael’s fcode**: *o1o1o1o1o1.
10. **Lamech (Methushael’s son)**.
    - Lamech is Methushael’s son, encoded as *o1o1o1o1o1o1 (Methushael’s first son).
    - **Lamech’s fcode**: *o1o1o1o1o1o1.

### **Final Fcodes:**

- **Adam**: *
- **Eve (Adam’s wife)**: *C.
- **Cain (Adam’s first son)**: *o1
- **Abel (Adam’s second son)**: *o2
- **Cain’s wife**: *o1C.
- **Enoch (Cain’s first son)**: *o1o1
- **Irad (Enoch’s son)**: *o1o1o1
- **Mehujael (Irad’s son)**: *o1o1o1o1
- **Methushael (Mehujael’s son)**: *o1o1o1o1o1
- **Lamech (Methushael’s son)**: *o1o1o1o1o1o1

### **Justifications:**

- **Adam**: As the origin of coordinates, Adam is represented by *.
- **Eve**: Eve, as Adam’s wife, is encoded as *C..
- **Cain**: As Adam’s first son, Cain is encoded as *o1.
- **Abel**: As Adam’s second son, Abel is encoded as *o2.
- **Cain’s wife**: Cain’s wife is encoded as *o1C..
- **Enoch**: As Cain’s first son, Enoch is encoded as *o1o1.
- **Irad**: As Enoch’s son, Irad is encoded as *o1o1o1.
- **Mehujael**: As Irad’s son, Mehujael is encoded as *o1o1o1o1.
- **Methushael**: As Mehujael’s son, Methushael is encoded as *o1o1o1o1o1.
- **Lamech**: As Methushael’s son, Lamech is encoded as *o1o1o1o1o1o1.

This set of Fcodes represents the genealogy of Adam’s family according to the information provided.

## Gemini Advanced 1.5 Pro (9/9)

**Adam:** *

**Eve:** *C. (Adam's wife, and we have no information about her siblings)

**Cain:** *o1 (Adam and Eve's first son)

**Abel:** *o2 (Adam and Eve's second son)

**Enoch (Cain's son):** *o1o1

**Irad (Enoch's son):** *o1o1o1

**Mehujael (Irad's son):** *o1o1o1o1

**Methushael (Mehujael's son):** *o1o1o1o1o1

**Lamech (Methushael's son):** *o1o1o1o1o1o1

# Prompt 9 - La Regenta (Chapter 4)

## Prompt

The Ozores family was one of the oldest in Vetusta. That surname belonged to many counts and marquises, and there were few nobles in the city who were not, in one way or another, somewhat related to such an illustrious lineage.

Don Carlos, Ana's father, was the eldest son of a younger branch of the Count of Ozores. Don Carlos had two sisters, Anunciación and Águeda (the youngest), who, along with their father, lived for a long time in the ancestral mansion. The main branch, that of the counts, had emigrated years ago.

The eldest son of the younger branch wanted a career, to be more than just the heir to some farmhouses, a few estates, and a dilapidated palace full of leaks. He became a military engineer. He proved his bravery in many battles; he demonstrated great knowledge in Vauban's art, built durable and well-designed forts on various coasts, and quickly rose to the rank of army colonel, commanding the corps.

[...]

Madly in love, Don Carlos Ozores married at the age of thirty-five a humble Italian seamstress who lived surrounded by countless temptations, yet remained honourable and poor. She was Ana's mother.

Using Ana Ozores as the origin of coordinates, write the Fcodes of her family.

## Expected result

* Ana Ozores

*M. Italian seamstress

*P1 Don Carlos Ozores

*P.P. Conde de Ozores

*P1A2 Anunciación Ozores

*PA3 Ágeda Ozores

## ChatGPT-4o (6/6) + 1

Using **Ana Ozores** as the origin of coordinates (denoted as *), let's break down the family relationships described in the story and generate the appropriate Fcodes based on her family structure.

### **Relationships and Justifications:**

1. **Ana Ozores (origin of coordinates)**.
   - Ana Ozores is the focal point, so her Fcode is *.
   - **Ana Ozores’s fcode**: *.
2. **Don Carlos (Ana’s father)**.
   - Don Carlos is Ana’s father, the eldest son of a younger branch of the Count of Ozores. Since no sibling order relative to his own siblings is specified, we will encode him as P. (father).
   - **Don Carlos’s fcode**: *P.
3. **Ana’s mother**.
   - Ana’s mother, a humble Italian seamstress, is Don Carlos’s wife. Since no sibling order is provided for her, we encode her as M. (mother).
   - **Ana’s mother’s fcode**: *M.
4. **Anunciación (Ana’s paternal aunt)**.
   - Anunciación is one of Don Carlos’s two sisters. Since Anunciación’s birth order relative to Don Carlos is not specified but is older than Águeda, we can encode her as the second sibling using A2 (second sister).
   - **Anunciación’s fcode**: *P.A2
5. **Águeda (Ana’s paternal aunt)**.
   - Águeda is Don Carlos’s youngest sister. Therefore, she is encoded as A3 (third sister).
   - **Águeda’s fcode**: *P.A3
6. **Ana’s paternal grandfather (the father of Don Carlos, Anunciación, and Águeda)**.
   - Ana’s paternal grandfather is not named but is described as having lived for a long time in the ancestral mansion along with his children. Since he is Don Carlos’s father, we encode him as P.P..
   - **Ana’s paternal grandfather’s fcode**: *P.P.
7. **The Count of Ozores (the head of the main branch of the family)**.
   - The Count of Ozores is the head of the main branch, but there is no direct familial connection specified between him and Ana, except that Don Carlos belongs to a younger branch of this family. Therefore, no specific Fcode is provided for the Count as he does not form part of the immediate family in this story.

### **Final Fcodes:**

- **Ana Ozores**: *
- **Don Carlos (Ana’s father)**: *P.
- **Ana’s mother (Don Carlos’s wife)**: *M.
- **Anunciación (Ana’s paternal aunt)**: *P.A2
- **Águeda (Ana’s paternal aunt)**: *P.A3
- **Ana’s paternal grandfather**: *P.P.

### **Justifications:**

- **Ana**: As the origin of coordinates, Ana is represented by *.
- **Don Carlos**: As Ana’s father, Don Carlos is encoded as *P..
- **Ana’s mother**: As Ana’s mother, she is encoded as *M..
- **Anunciación**: As Ana’s father’s second sister, Anunciación is encoded as *P.A2.
- **Águeda**: As Ana’s father’s youngest sister, Águeda is encoded as *P.A3.
- **Ana’s paternal grandfather**: As Ana’s paternal grandfather, he is encoded as *P.P..

This set of Fcodes represents the genealogy of Ana Ozores’s family according to the information provided in the story.

## Gemini Advanced 1.5 Pro (2/6)

**Ana Ozores:** *

**Don Carlos Ozores (Ana's father):** *P1 (Eldest son of a younger branch)

**Ana's Mother:** *P1C. (Married to Don Carlos, no information on her birth order)

**Anunciación Ozores (Ana's aunt):** *P1A1 (Older sister of Don Carlos)

**Águeda Ozores (Ana's aunt):** *P1A2 (Younger sister of Don Carlos)

# Prompt 10 - The Bourbon Dynasty (historyofspain.es)

## Prompt

Carlos III, who was the son of Philip V and his second wife Isabel of Farnesio, came to the throne.

The successor of Carlos III was his son Carlos IV, known as “the Hunter”. He was not very involved in government matters as King, and he largely left such things in the hands of his wife Maria Luisa of Parma.

After the mutiny of Aranjuez, Carlos IV had to abdicate in favour of his son Fernando VII.

Using Maria Luisa of Parma as the origin of coordinates, write the Fcodes of the people in this story.

## Expected result

* Maria Luisa of Parma

*C. Carlos IV

*C.P. Carlos III

*o. / *o1 Fernando VII

*C.P.P. Philip V

*C.P.M. Isabel of Farnesio

## ChatGPT-4o (6/6)

Using **Maria Luisa of Parma** as the origin of coordinates (denoted as *), let's break down the relationships described in the story and generate the appropriate Fcodes based on the family structure.

### **Relationships and Justifications:**

1. **Maria Luisa of Parma (origin of coordinates)**.
   - Maria Luisa of Parma is the origin of coordinates, so her Fcode is *.
   - **Maria Luisa’s fcode**: *.
2. **Carlos IV (Maria Luisa’s husband)**.
   - Carlos IV is Maria Luisa’s husband. Since no sibling order is provided for him, we will encode him as C. (spouse).
   - **Carlos IV’s fcode**: *C.
3. **Fernando VII (Maria Luisa’s son)**.
   - Fernando VII is the son of Maria Luisa and Carlos IV. He succeeded his father as king, making him the eldest son, so he is encoded as o1 (first son).
   - **Fernando VII’s fcode**: *o1.
4. **Carlos III (Maria Luisa’s father-in-law)**.
   - Carlos III is Carlos IV’s father, making him Maria Luisa’s father-in-law. He is encoded as C.P. (father of Maria Luisa’s spouse).
   - **Carlos III’s fcode**: *C.P.
5. **Philip V (Carlos III’s father)**.
   - Philip V is the father of Carlos III, making him Maria Luisa’s spouse’s paternal grandfather. He is encoded as C.P.P. (father of Carlos III, the spouse’s grandfather).
   - **Philip V’s fcode**: *C.P.P.
6. **Isabel of Farnesio (Maria Luisa’s spouse’s paternal grandmother)**.
   - Isabel of Farnesio is Carlos III’s mother, making her Maria Luisa’s spouse’s paternal grandmother. She is encoded as C.P.M. (mother of Carlos III).
   - **Isabel of Farnesio’s fcode**: *C.P.M.

### **Final Fcodes:**

- **Maria Luisa of Parma**: *
- **Carlos IV (Maria Luisa’s husband)**: *C.
- **Fernando VII (Maria Luisa’s son)**: *o1
- **Carlos III (Maria Luisa’s father-in-law)**: *C.P.
- **Philip V (Carlos III’s father)**: *C.P.P.
- **Isabel of Farnesio (Carlos III’s mother)**: *C.P.M.

### **Justifications:**

- **Maria Luisa**: As the origin of coordinates, Maria Luisa is represented by *.
- **Carlos IV**: As Maria Luisa’s husband, Carlos IV is encoded as *C..
- **Fernando VII**: As Maria Luisa’s first son, Fernando VII is encoded as *o1.
- **Carlos III**: As Maria Luisa’s father-in-law, Carlos III is encoded as *C.P..
- **Philip V**: As Carlos III’s father and Maria Luisa’s spouse’s paternal grandfather, Philip V is encoded as *C.P.P..
- **Isabel of Farnesio**: As Carlos III’s mother and Maria Luisa’s spouse’s paternal grandmother, Isabel of Farnesio is encoded as *C.P.M..

This set of Fcodes represents the family members connected to Maria Luisa of Parma as described in the story.

## Gemini Advanced 1.5 Pro (4/6) - 2

You got it! Here are the fcodes using Maria Luisa of Parma as the origin of coordinates:

- **Maria Luisa of Parma:** *
- **Carlos III (her husband's father):** *C.P. (We don't know Carlos III's birth order among his siblings)
- **Carlos IV (her husband):** *C. (We don't know Carlos IV's birth order among his siblings)
- **Fernando VII (her son):** *o1 (Assuming he's her oldest son, as no other children are mentioned before him in this text)

**[PROMPT]: *Add the Fcodes of Isabel of Farmesio and Philip V***

Here's the updated list with the added fcodes:

- **Maria Luisa of Parma:** *
- **Carlos III (her husband's father):** *C.P.
- **Isabel of Farmesio (her husband's mother):** *C.M.
- **Carlos IV (her husband):** *C.
- **Philip V (her husband's father):** *C.P.
- **Fernando VII (her son):** *o1
